# Alleviation of Tumor Invasion by the Development of Natural Polymer-based Low-risk Chemotherapeutic Systems – review on the Malignant Carcinoma Treatments

**DOI:** 10.2174/0115672018349688241008220007

**Published:** 2024-10-14

**Authors:** Vignesh Natarajan

**Affiliations:** 1 School of Biosciences Engineering and Technology, Vellore Institute of Technology Bhopal University, KothriKalan, Bhopal 466114, India

**Keywords:** Polysaccharide, liposome, peptide, colon carcinoma, breast carcinoma, chemotherapy

## Abstract

**Introduction/Objective:**

The spread of tumors (48% in men and 51% in women), as well as the protection of malignant tumors by stromal cells and complex blood vessels, pose significant challenges to drug delivery to tumors. Modern chemotherapy, on the other hand, addresses tumor growth suppression by at least 60% through versatile formulation systems and numerous modifications to drug delivery systems. The renewable and naturally occurring polymers present invariably in all living cells form the fundamental foundation for most anticancer drug development. The review aims to discuss in detail the preparations of polysaccharide, lipid, and protein-based drug-loading vehicles for the targeted delivery of prominent anticancer drugs. It also provides an explanation of drug distribution in blood (cumulative releases of nearly 80% drug) and drug accumulation at tumor sites (1–5 mg/kg) due to enhanced permeability and retention (EPR).

**Methods:**

Specific delivery examples for treating colorectal and breast carcinomas have been presented to distinguish the varied drug administration, bioavailability, and tumor internalization mechanisms between sugar, fatty acid, and amino acid polymers. Current therapy possibilities based on cutting-edge literature are provided, along with drug delivery systems tailored to tumor location and invasive properties.

**Results:**

The unique combinations of the three natural polymers provide unparalleled solutions to minimize the toxicity (<20% drug release) of the chemotherapeutic drugs on normal tissues. Moreover, the development of a consolidated drug delivery system has contributed to a substantial reduction (dose reduction from 10.43 µM to 1.9 µM) in the undesirable consequences of higher dosages of chemotherapeutic drugs.

**Conclusion:**

The review extensively covers safe chemotherapeutic systems with significant advantages (tumor volume shrinkage of 4T1 cells from 1000 mm^3^ to 200 mm^3^) in clinical applications of carcinoma treatments using natural polymers.

## 1 INTRODUCTION

Understanding cell division, a process that involves the equal or undetermined partitioning of both cytoplasmic content and the genetic material, is crucial in the context of cancer. This complex process has several checkpoints that control the population of ectoderm-, mesoderm-, and endoderm-derived cells [1]. Embryonic development and wound healing, for instance, require actively dividing cells to produce multicellular organisms and regenerate tissue, respectively. However, acquiring physiological properties comes with cell differentiation, which provides distinct functions for numerous cell types such as red blood cells, fibroblast, neurons, hepatocyte, intestinal epithelial cells, osteoblasts, *etc*. [2]. Cancer reverses this process, causing cells to undergo immortalization, escape apoptosis, and produce rapidly proliferating cells with intense metabolic requirements [1].

Also, these cells are very invasive when they move to faraway body parts through the circulatory system. This implies a significant likelihood of tumor growth in the body [3]. The causes of cancer are innumerable, and the primary treatment for elimination is surgical removal of tumor growth. In addition to surgical intervention, a nearly complete eradication of cancer warrants the controlled killing of tumor cells by either radiation therapy, chemotherapy, or a combination of both [4]. Radiation therapy, by and large, is associated with risks of genetic mutations, neuron damage, hair loss, and other unpredictable long-term dire consequences. As a result, people seek chemotherapy as a safer alternative to radiation therapy, but the random cytotoxicity of chemotherapeutic drugs necessitates the careful development of a drug delivery system [5]. The classification of the drugs used in chemotherapy includes alkylating agents (nitrosoureas), mitotic inhibitors (paclitaxel, docetaxel), intracellular reactive oxygen species inducers (doxorubicin, anthracycline groups of antibiotics derived from Streptomyces species), topoisomerase inhibitors (camptothecin, cisplatin), antimetabolites (methotrexate), and angiogenic inhibitors (bevacizumab) [6]. Chemotherapeutic drugs must be bioavailable for a longer time in the bloodstream, less likely to leak out into the surrounding area, more likely to build up at the tumor site, and very specific in their release (either directly into cells or through the immune system) to avoid side effects and immune system reactions. It is important to make drug delivery systems that release drugs in response to physiological signals [7]. This is because drugs, unlike water, have different molecular structures and target different things, such as receptors on the plasma membrane, DNA, cytosolic proteins, and extracellular proteins [8].

Moreover, the physiology of tumors is largely distinct and provides critical conditions for drug carrier development [3]. Some delivery systems depend on charge interactions, the change from hydrophobic to hydrophilic, amphiphilic properties, and self-assembly. Other systems use covalent bonds, metal ion complexation, and other things [8]. However, the therapeutic window of the chemotherapeutic drug against tumors is limited. More than the expected outcome of tumor regression, the tissues with abundant vascularization, such as the lung, kidney, and liver, are vulnerable to drug toxicity [9]. Researchers are writing more and more studies that focus on tumor cytotoxicity by choosing nanocarrier drug delivery systems and using either single-agent or combined approaches [10-12]. Natural polymers, in contrast to synthetic polymers, have found widespread application in the development of drug delivery systems. Using natural polymers offers numerous advantages, such as their ease of synthesis, their ability to interact effectively with cells and the host system, their minimal impact on the immune system, their ability to interact effectively with ligands and receptors, their ability to produce less harmful intermediate metabolites, and many others [13]. Researchers broadly classify the natural polymers that make up a cell (a biological entity) into carbohydrates, proteins, and lipids, from which we derive numerous extracellular products [14]. Natural polymers, isolated in their polymeric form either from plants or algae (starch, alginate, cellulose) or animals (chitosan, hyaluronic acid, and gelatin), are associated with sustainable and facile extraction methods. In a few cases, monomers produced by microbial fermentation have been polymerized into biodegradable material (polylactic acid) [15]. In contrast, synthetic polymers (polycaprolactone), which are non-renewable in origin and mostly petroleum-derived, have limitations such as waste management, extensive carbon emissions, soaring market prices, depleting crude oil reserves, and so on. Even though there have been batch-to-batch variations, seasonal variations, and slow processing of natural polymers, these are integral parts of all living cells and hence highly suitable for drug delivery applications due to their immense biocompatibility [16]. This review will provide comprehensive information on the drug delivery systems that utilize polysaccharides, lipids, and proteins to combat tumor growth and progression. Furthermore, specific cases of colon carcinoma and breast cancer have presented some fundamental mechanisms of drug delivery using the aforementioned natural polymers. The paper also elaborates on recent approaches that use a combination of polysaccharides, lipids, and peptides to treat tumor metastasis.

## 2 NATURAL POLYMERS IN THE FORMULATION OF DRUG DELIVERY SYSTEMS

### 2.1 Polysaccharide-based Drug Delivery System

The sugars, with their unique properties, offer a promising avenue for constructing a drug delivery system. Because they are simple to make and have a lot of hydroxyl groups for chemical modification, cytocompatibility, and other benefits, polysaccharides are the best way to deliver drugs with a wide range of physicochemical properties. Given cancer cells' internal biochemical metabolism [17], delivering polysaccharide-based drugs to tumors is a promising approach. The direct requirement of sugars to support tumor progression helps to circumvent the limitations of tumor stroma and complex blood vessels. The fact that sugars required for cellular metabolism are neutral further bolsters this optimistic outlook on the potential of polysaccharide-based nanocarriers [18].

Charged polysaccharides, on the other hand, are important in drug formulation development due to the availability of multiple drug interaction mechanisms (Table **1**). The polysaccharides, depending on their charge, exhibit pH-responsive activity [19]. For example, chitosan, positively charged at pH 6.5, binds strongly to negatively charged RNA and releases it in the tumor environment when pH falls to 4.5 [20]. Cyclodextrin releases drugs in a manner akin to ping-pong, where the water-repellent drug attaches to an inner core that remains uncharged at physiological pH. The change in pKa in the tumor microenvironment makes electrostatic repulsion easier, which then releases the drug slowly at the target site [21]. Scientists have made some intriguing amphipathic block copolymers using chemical grafting and free-radical polymerization to improve how drugs interact with each other and how they move through the bloodstream [22]. However, additional steps of tumor accumulation, penetration into tumor tissues, and internalization by tumor cells limit the drug delivery to the tumor [19].

Moreover, drug administration becomes more challenging as metabolic requirements and tumor tissue architecture alter with each stage of tumor growth [23]. In the initial hypoxic state, cells need to be able to take in glucose without any problems. As a result, the metabolism changes to aerobic glycolysis, new blood vessels grow, and the extracellular matrix thickens. This makes it easier for metabolites to move around inside the cancer cell [24]. There is a transition in the tumor microenvironment during the transformation of benign adenomas into malignant neoplasm, causing subtle alterations in the extracellular matrix [25]. As the tumor gets bigger, the drug works less well, and the polysaccharide-based nanocarriers have to deal with complicated microenvironments before they can reach the cancer cell [23]. Hence, the design of polysaccharide-based nanocarriers has to be tailored depending on the tumor microenvironment and potential physiological barriers that inhibit the targeted delivery of anticancer drugs [19].

The leaky vasculature of blood vessels around tumor tissue is crucial for drug delivery because the molecular size and sieving properties make it easier for drugs to get into the tumor [26]. Important proteins like angiotensin and angiopoietin do not express as much during angiogenesis, resulting in larger pores in blood vessels. These proteins control the structure and overall architecture of endothelial cells in capillaries [27]. Passive targeting occurs when nanoparticles gather in the tumor's blood vessels. This occurs because of the sieving effect, which aids in EPR [26]. Polysaccharides form the basis of this type of nanocarrier. It needs to have diameters of about 100 nm to stop passive diffusion across normal blood vessels and allow selective permeation in the tumor vasculature. However, the lymphatic system rapidly clears the smaller nanoparticles in the tumor microenvironment, which minimizes tumor accumulation and drug efficacy [28]. Scientists have created effective nanomaterials capable of self-assembly, forming groups that adapt to the tumor's surroundings and resist cell flushing. When attached to hyaluronic acid, the anticancer drug paclitaxel from the Pacific Yew tree triggered the self-assembly of nanoparticles [29]. The aerobic glycolytic pathway in the tumor cells produced excessive lactate. This caused the gold nanoclusters covered in sodium alginate to stick together quickly in the acidic area around the tumor [30]. Adding an amphiphilic block copolymer changes the polysaccharides in a way that protects them from glycosidases. It also increases the particle size in the delivery system so that they can only pass through the endothelial junctions in the blood vessels that supply the tumor [31]. By connecting chondroitin sulfate and retinoic acid, Li *et al*. (2022) created the prodrug, which is a building block of an active drug ingredient that fights the metastasized tumor. It is an amphiphilic block copolymer. When it interacts with the CD44 receptor ligand, the tumor cell receives paclitaxel [32]. Fig. (**1a**) illustrates that sialic acid, present on the surface of tumors, facilitates drug entry into cells by interacting with the CD44 receptor [33].

**Fig. (1):**
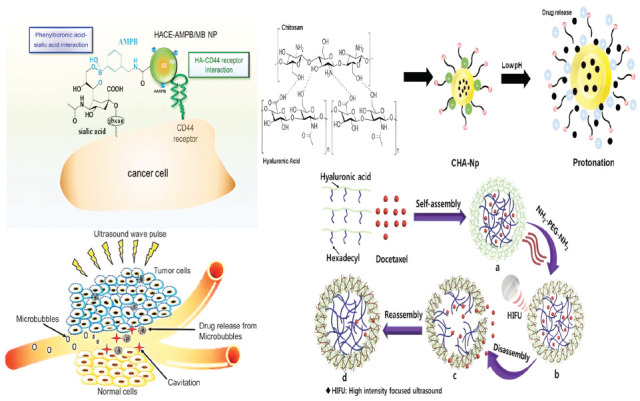
**(a)** Passive tumor targeting through hyaluronic acid interaction with CD44 receptor and binding of AMPB with tumor marker sialic acid proteoglycan on cancer cell (Reproduced with copyright permission from [33], Elsevier) **(b)** Ultrasound induced enlargement of capillary junction and enhanced permeation and retention effect in cancer cells (Reproduced with copyright permission from [36], Elsevier) **(c)** Development of polyelectrolytes consisting of positively charged chitosan and negatively charged hyaluronic acid to deliver the drug under acidic pH (Reproduced with copyright permission from [42], MDPI journal) **(d)** Ultrasound regulated release of docetaxel through cavitation induced disassembly and spontaneous reassembly of hexadecyl modified hyaluronic acid (Reproduced with copyright permission from [46], Elsevier).

Nevertheless, the entry into the tumor stroma is not the final step, and the uptake of drugs by tumor cells determines the drug delivery system's anticancer activity. Once again, large drug molecules impede tumor cells' entry, and they play a crucial role in dissolving the aggregate structure into nanoparticles, enabling the drugs to penetrate the tumor cell effectively [34]. When used together, the ultrasound precursors perfluoropentane and fluorescein isothiocyanate (FITC)-tagged folate helped create microbubbles at body temperature and widen blood vessels through cavitation, increasing the likelihood of folate interacting with tumor cell receptors [35]. The stimuli-responsive nanocarriers help the drug get into the right places in the body in a number of ways (Fig. **1b**) [36]. Studies have demonstrated that using ultrasound to break up the polysaccharide-based nanocarrier system enhances the drug's absorption [37, 38]. A few cases report the negatively charged polysaccharides as excellent ligands for specific markers and receptor proteins in the tumor cells. For instance, the expressed CD44 receptor in B-cell lymphoma effectively recognizes hyaluronic acid [39].

Besides, hyaluronic acid is associated with reduced susceptibility to clearance by the host reticuloendothelial system and opsonization [19]. The carboxyl, N-acetyl, and hydroxyl groups of hyaluronic acid allow for numerous chemical changes. Hyaluronidase, on the other hand, breaks it down in the area around a tumor [40]. Luan *et al*. (2023) created a drug delivery system with hyaluronic acid on the outside and amphiphilic hexadecapeptide on the inside. This allowed to control how easily the anticancer drug doxorubicin could enter the tumor [41]. On the other hand, the mixture of metal ions and sodium alginate transforms it from a sol to a gel. It is one of the best ways to deliver drugs through the tumor's blood vessels and microenvironment [19].

However, polysaccharide-based systems require the presence of more than a polymer to increase the therapeutic efficacy [37]. Mixing a negatively charged polysaccharide with a hydrophobic, neutral, or positively charged polysaccharide (Fig. **1c**) facilitates the tumor's growth and entry into the cell [21, 31, 42]. In order to make sure that doxorubicin, a hydrophobic drug, gets to the right place in neuroblastoma cancer cells, researchers attach polysaccharides like cyclodextrin and hyaluronic acid [43]. Sui *et al*. (2020) used an acid-cleavable hydrazone bond. This made the chemotherapy drug work better on the MCF-7 breast cancer cell line, which is resistant to many drugs. Additionally, the disulfide bond between hyaluronic acid and pullulan facilitated the uptake of doxorubicin through CD44-mediated endocytosis [44]. Adding a photosensitizer to the hyaluronic acid-cyclodextrin hybrid polymer made it easier for the drug to reach the right tumors and increased its effectiveness by 35%. This was because light caused the hyaluronic acid and CD44 receptors in breast cancer to interact with each other [45]. Fig. (**1d**) illustrates how ultrasound controls the assembly and dissolution of hexadecylated hyaluronic acid to deliver doxorubicin [46]. Nanoparticles are also added to the sugar polymers to change how they react to different stimuli. This ensures precise drug delivery to the tumor tissue *via* light, ultrasound, or pH. In other words, the nanoparticles channel highly deleterious chemotherapeutic drugs into a very narrow distribution only in the tumor microenvironment. New research, for instance, explores the potential of combining physical (external) and physiological (internal) stimuli to enhance the pharmacokinetics of polysaccharide-based nanocarrier systems [19]. Researchers treated people with pancreatic cancer by selecting the appropriate cargo with a light-emitting chemical. This created reactive oxygen species and made the chitosan-based nanosystem more permeable. When nanoparticles were put under 0.5 W/cm^2^ of light and zinc phthalocyanine, a photosensitizer, was added, the EPR effect became much stronger [47]. The treatment of diffuse intrinsic pontine glioma with doxorubicin is obsolete without special measures to increase the drug permeability across the blood-brain barrier. Convection-enhanced delivery (CED), an invasive technique, typically shrinks the tumor by inserting a catheter into the brain stem and maintaining a high-pressure gradient [48]. Researchers have discovered a way to use focused ultrasound guided by magnetic resonance imaging to deliver a nanodroplet containing doxorubicin, which is made of alginate and dopamine and responds to ultrasound. This slows the growth rate of the tumor volume by changing the blood-brain barrier in mice with diffuse intrinsic pontine glioma. Furthermore, the limited distribution of doxorubicin in the tumor tissue was validated to prevent the drug’s non-specific cardiotoxicity effects and nullify the multiple drug resistance (MDR) emergence [49].

### 2.2 Liposome

**Fig. (2):**
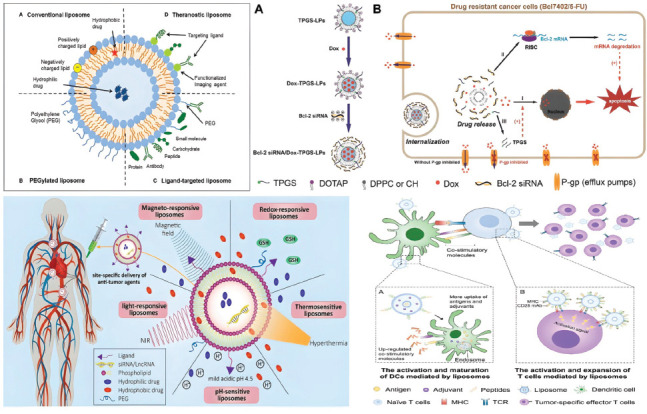
**(a)** The overall functionalization of liposome based nanocarrier with polyethylene glycol, amphiphilic drug, imaging agent, carbohydrate, peptide, monoclonal antibody (Reproduced with copyright permission from [56], MDPI journal) **(b)** Co-delivery of siRNA with chemotherapeutic drug doxorubicin using cationic liposome DOTAP (Reproduced with copyright permission from [59], Elsevier) **(c)** Site specific delivery combined with stimuli responsive release for effective chemotherapeutic treatment using liposomal nanovesicle (Reproduced with copyright permission from [60], Elsevier) **(d)** Immunomodulatory effects of liposomes in eliciting both humoral and cell-mediated immune responses against cancer progression (Reproduced with copyright permission from [64], MDPI journal).

Lipids, triglycerides, phospholipids, and cholesterol form the universal physiological barrier separating the intracellular and extracellular environments in all prokaryotes and eukaryotes. Hence, lipids are undoubtedly the innate biocompatible polymers for developing drug delivery systems [50]. Liposomes are formed by two layers of lipids in an aqueous environment. They are a very flexible way to load and deliver drugs that are hydrophobic, hydrophilic, or amphiphilic (Table **1**). Hence, liposomes are considered to be multifunctional drug delivery systems [51]. Liposomes are made up of phosphatidyl choline, phosphatidyl ethanolamine, and phosphatidyl glycerol. Scientists have used these to make micelles and dendrimers that hold drugs that fight cancer [52]. Phospholipids are useful because they can add charged species to polar head groups. It has been possible to connect phospholipids to antibodies, RNAs, and aptamers through graft copolymerization, click chemistry, and disulfide linkages [53]. Unlike polysaccharide-based systems, the reticuloendothelial system gets rid of liposomes quickly, and phagocytes break them down through opsonization. The linking of polyethylene glycol (PEG) to the charged head group in phospholipid has kept the liposomes safe, stopping them from breaking down and making them more bioavailable [54]. PEGylated liposomes and chitosan, hydroxyethyl starch, and sodium alginate-based nanocarrier systems all work about the same way when it comes to distributing drugs [50]. Moreover, liposomes can bind to multiple drugs, regardless of their hydrophobicity, and possess highly tumor-specific antibodies that enable them to traverse the diverse extracellular barriers in the tumor microenvironment (Fig. **2a**), ultimately reaching the tumor cell with remarkably high rates of accumulation and internalization [55, 56]. Still, liposomes are stable and have a varied makeup, which makes it harder to actively target them when making a multifunctional drug delivery system. It is impossible to stop the drug from passively diffusing from the liposome to the extracellular space without adding sterol groups to the lipid bilayer [50]. Cholesterol is a more concentrated form of squalene. It has denser aromatic rings and hydroxyl functional groups that pair with triglycerides and phospholipids to make it harder for smaller molecules to move across the lipid bilayer. Adding different kinds of lipids requires a thorough study of the drug's stability, distribution, and therapeutic effectiveness in living organisms, along with validation using a wide range of statistical tools [57]. While the construction of a liposome necessitates the optimization of multiple factors, a single liposome functions as a payload vehicle with a cascade drug delivery system to slow the progression of metastatic solid tumors [50, 51]. Researchers have found that the ligand CXC chemokine receptor type 4 helps breast tumors grow by combining its function with the movement of cells in tumor metastasis. Gote & Pal (2021) reported critical inhibition of metastasis by designing a liposomal system to not only deliver the antagonist CXCR4 to cause receptor inactivation but also release the small interfering RNA drug (Fig. **2b**), lipocalin 2, to prevent the epithelial-to-mesenchymal transition [58, 59]. By tailoring the liposomal composition and attaching a variety of cargo molecules, researchers have reported extremely site-specific drug delivery for breast cancer treatment (Fig. **2c**) [60]. For example, researchers fine-tuned the delivery of doxorubicin to accumulate at the tumor site by conjugating the p12 peptide with the stealth liposome and demonstrated the light-responsive drug release after tumor accumulation through the photothermal effect of tagged indocyanine green [61]. Researchers have extensively studied the monoclonal antibody-grafted liposome, a type of immunoglobin. It helps with drug delivery and the active targeting of antitumor drugs. Moreover, researchers have inserted the paratope regions of the monoclonal antibodies into the liposome to decrease opsonization and eliminate the specific interaction of the constant C-terminal region with the receptors of innate immune cells, such as macrophages and natural killer cells [50]. Some antibody preparations against tumor cell receptors have been straightforward, involving the saline extraction of monoclonal or antinuclear antibodies from the tumor cell. For example, tumor progression involves immortalization, in which cells undergo transient apoptosis and revert to malignant neoplasm in the later stages. During apoptosis, the extracellular space releases antinuclear antibodies that are bound to double-stranded DNA. These antibodies can be taken out and used to create the liposome delivery system [62, 63]. In other cases, T lymphocyte-activating antigens functionalize the liposomes carrying the anticancer drug doxorubicin, initiating a combination of chemotherapy and immunotherapy to suppress tumor metastasis and invasion (Fig. **2d**) [64]. For instance, researchers combined the docetaxel drug, which operates against erythropoietin-producing hepatocellular carcinoma receptors (EphA2), with anti-T-lymphocyte-associated protein 4-antibodies in a single PEGylated (stealth) liposome to reduce the severity of breast cancer progression by 60% [65]. The addition of cis-linoleic acid to paclitaxel extended its plasmatic half-life without altering its distribution in the glioblastoma site or ability to cross the blood-brain barrier [66]. Blood vessel formation intimately connects with the tumor's progression, and the key protein involved in angiogenesis is the vascular endothelial growth factor. Hypoxia triggers factors that increase VEGF production. This, in turn, is linked to the amount of reactive oxygen species in the tumor cell [3]. Dong *et al*. (2023) combined the drugs camptothecin and α-tocopherol, which can stop topoisomerase and remove reactive oxygen species, with a nanoparticle drug delivery system made up of tetraethylene glycol and lipoic acid to stop tumors from spreading and advancing [67]. Nel *et al*. (2023) investigated the importance of delivering an anticancer drug *via* lipid to evade the ABC transport protein involved in drug efflux and discovered that surfactants such as polysorbate 80 increased drug uptake by tumor cells without affecting protein expression profiles. One important step in stopping ABC transport, or Pgp protein [50], is making sure that the drug can enter the cell through the liposome without needing ATP. The tumor cell evades the host immune system in multiple ways. One important mechanism is the expression of a changed major histocompatibility complex class I, which carries an antigen specific to the tumor and prevents natural killer cells from contacting and killing it [4]. Using the same method, the research group made a PEGylated liposome with an antigen from the glioma membrane. This increased the drug's distribution and plasmatic half-life, as well as guided the liposome to glioblastoma in a mouse model [66]. A small number of studies have shown that passive targeting of liposomal nanocarriers alone is necessary to get many chemotherapeutic drugs to cancerous tumors. However, liposome functionalization is essential to combat the drug clearance system while also facilitating drug uptake [50]. With the help of a cationic liposomal vehicle, Li and his colleagues (2022) delivered two chemotherapeutic drugs, paclitaxel and crizotinib, along with small interfering RNA, to MCF-7 cells. The purpose was to provide nanocarrier with amphiphilic property to encapsulate hydrophilic paclitaxel and negatively charged siRNA in the aqueous core, as well as embed the hydrophobic crizotinib in the lipid bilayer. When DOPE and DOTAP were used to make liposomes, it was easier for the positively charged head groups to bind to siRNA in the center and the tumor membrane on the outside. Despite demonstrating the synergistic effects of siRNA and chemotherapeutic drugs on the downregulation of antiapoptotic protein Bcl-X2 and cell cycle arrest, respectively, the study limited its evaluation to *in vitro* conditions and did not assess the pharmacokinetics of liposomes *in vivo* [68].

### 2.3 Peptide-based Nanocarrier

**Fig. (3):**
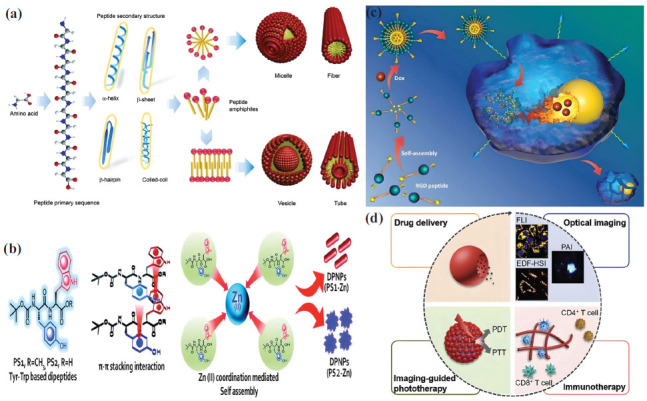
**(a)** Mechanism of self-assembly of peptide amphiphiles into nanomicelle, nanofiber, nanotube and nanovesicle (Reproduced with copyright permission from [75], Royal Society of Chemistry) **(b)** Zinc ion induced self assembly of tyrosine-tryptophan dipeptide (Reproduced with copyright permission from [77], Royal Society of Chemistry) **(c)** pH dependent release of drug doxorubicin from LKR cyclic peptide and RGD sequence mediated drug uptake by tumor cell (Reproduced with copyright permission from [81], Elsevier) **(d)** Applications of peptide-based nanocarriers in cancer diagnosis and treatment (Reproduced with copyright permission from [82], Cell Press).

Peptides with different lengths from the native protein are crucial for making new drugs and delivering them specifically to tumors (Table **1**). The selection of protein sequences for drug delivery has many advantages, but the principal benefit is the alteration of extracellular factors that contribute to tumor regression. Because of this, the drug delivery system's diffusion limitation and effective intracellular uptake are no longer issues that stop tumor growth or make it much slower [69]. It has been done using monoclonal antibodies to make peptides that target tumor-promoting factors such as matrix metalloproteinases (MMPs), vascular endothelial growth factors (VEGF), angiotensin, and platelet-derived growth factor receptor (PDGFR) [70]. One major difference between peptide-based nanocarriers and other drug delivery systems is that the peptides naturally help shrink tumors, which works with the chemotherapeutic drug [71]. More importantly, chemotherapeutic drug's effective dosage value has been lowered to reduce toxicity levels and prevent future non-targeted effects [72]. Even though peptide-based systems can be made in a lab, studies have shown that naturally occurring endotoxins can stop extracellular matrix proteins and integrins from working. However, these proteins have high toxicity and diminished selectivity [73]. There are two types of lipoproteins: those that attach proteins to phospholipids and those that may be able to move peptide-based nanocarriers through the bloodstream [74]. Many factors contribute to tumor formation, but extracellular factors, in particular, govern the tumor's metastasis [3]. Even though peptide-based nanocarriers are associated with restricted diffusivity across the tumor cell membrane, these are essential to reduce tumor invasion [70]. When it comes to non-covalent interactions between molecules, peptides have more freedom than full-length proteins. Stacking, hydrophobic, electrostatic, and hydrogen bonding are the prominent interactions that aid in the self-assembly of peptides (Fig. **3a**) [75]. Unlike the body's natural amino acids, drug delivery systems primarily synthesize peptides artificially using specific, rationally prepared R or side chain groups [76]. For example, covalent modification of two polar amino acids, namely glycine, and alanine, into diphenyl glycine and diphenyl alanine enhances the binding of hydrophobic drugs and creates an amphiphilic environment. The peptides synthesized using diphenyl glycine were self-assembled into spherical nanostructures. Diphenyl alanine-containing peptides formed nanotubes [70]. The self-assembled structure's geometry, the active pharmaceutical ingredient's nature, and the drug delivery system's application against tumor change all play a role. For instance, the intravenous administration of hydrophobic doxorubicin using the zinc ion cross-linked tyrosine-tryptophan dipeptide nanostructure demonstrated the high efficacy of the peptide system against the lung cancer epithelial cell line (A549), as shown in Fig. (**3b**) [77]. In the case of 5-fluorouracil and methotrexate, which are inherently hydrophilic, the encapsulation and drug release patterns improved using the self-assembled D-leucine-phenylalanine tripeptide repeat, forming the supramolecular nanostructure [78]. In a murine model, the onsite delivery of doxorubicin using the hydrogel produced by the self-assembly of carboxy-termini-modified alanine peptide was demonstrated to treat solid tumors [79]. One of the most common peptide-based systems that binds very specifically to integrin receptors is made up of nine amino acids that are amphiphilic and form a cyclic peptide structure [70]. Cyclization has reduced surface charges and zeta potential, promoting hydrophobic interactions between the peptide-based nanocarrier and the plasma membrane. Furthermore, studies have reported that cyclic peptide-based systems can withstand proteases and enhance the drug's bioavailability [80]. Gong *et al*. (2021) made an amphiphilic pH-responsive peptide nanocarrier by putting together the RGD sequence with lysine, which is hydrophilic, and leucine, which is hydrophobic (Fig. **3c**) [81]. The peptides self-assembled into spherical nanoparticles with hydrophobic centers under physiological pH, enabling the encapsulation of doxorubicin without the need for covalent bonds. Even so, the acidic pH of the tumor microenvironment sped up the opening of the ring of the peptide-based nanocarrier by adding a proton to the ɛ-amino group of lysine. As a result, doxorubicin was released into mouse hepatoma cells (H22) and HepG2 cells *in vivo* with a high degree of specificity and αυβ3 integrin targeting. This enhanced the burst release of doxorubicin into mouse hepatoma cells [81].

Peptide-based delivery is unique in that it provides an additional flexible platform for real-time monitoring and imaging of pharmacokinetics (Fig. **3d**) [82]. To identify tumor accumulation and drug internalization, fluorescence shift properties were modulated using metal ion coordination chemistry [70]. For instance, when the cyclic dipeptide tyrosine-tryptophan bound to the Zn^2+^ ion, it changed its emission wavelength dramatically, going from ultraviolet to visible [77]. Additionally, cyclic L-histidine and D-histidine, when enclosed with epirubicin, transported the drug into the nucleus of the HeLa cell line, altering its emission wavelength from 390 nm to 500 nm. This was caused by the formation of a hexamer coordination complex with zinc ions [83]. The plasma membrane contains integral membrane proteins, which are highly effective in identifying tumors in specific tissues. There are three parts: the extracellular domain (N-terminal), the central hydrophobic transmembrane domain, and the cytosolic domain (C-terminal). The N-terminal domain of the receptor is the first part that can be accessed. Manipulating amino acids in the peptide-based nanocarrier to recognize different N-terminal regions provides a multifunctional drug delivery system capable of interacting with more than one tissue-specific surface marker of a tumor cell [84]. For instance, the CCL5 and CXCR4 chemokines show a high affinity for binding with CXCL12 receptors expressed in myeloma and breast carcinoma, respectively. The exchange of chemokines' N-terminal domains alters receptor specificity. Computational methods alter the chemotherapeutic drug's receptor sequence to increase its effectiveness in the body, enabling it to bind to multiple types of cell surface receptors on a specific tumor cell [85].

## 3 NATURAL POLYMER-BASED DRUG DELIVERY SYSTEM FOR THE TREATMENT OF MALIGNANT TUMORS

Various stages describing the drug distribution begin with the drug administration route and progress to the blood plasma concentration, partitioning coefficient into the tumor site, diffusivity across the extracellular matrix, and intracellular concentration in a specific tumor cell. Pharmacokinetics is a modeling approach that depicts the complete drug distribution profile in separate entities known as compartments, with numerous linking factors promoting forward and reverse reactions [86]. The examples of doxorubicin distribution employing three alternative drug delivery systems, namely polysaccharide-, lipid-, and peptide-based systems, are examined in detail regarding colon carcinoma and breast cancer therapies. The selection of tumor types was based on the degree of malignancy and magnitude of prevalence in the current global population. More importantly, the precursor for both carcinomas is epithelial cells, and extensive literature studies have shown that epithelial tissue is not only associated with extreme metastatic potential but also forms the foundation for the emergence of multiple cancer cells in other tissues (osteosarcoma, lung carcinoma, leukemia, hepatoma, *etc*.) [87-90]. Hence, treating colorectal and breast cancers provides adequate therapeutic measures to alleviate the complications of metastatic tumors and eliminate the risk of cancer recurrence [91, 92].

### 3.1 Drug Delivery for Colon Carcinoma Treatment

**Fig. (4):**
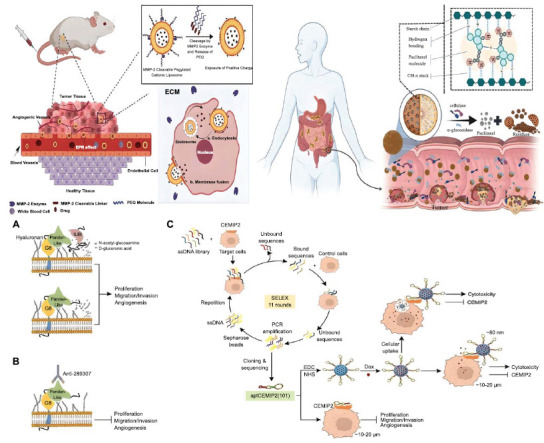
**(a)** Delivery of doxorubicin to C26 colorectal cancer cell through matrix metalloproteinase 2 induced surface changes on cationic liposome (Reproduced with copyright permission from [95], BioMed Central journal) **(b)** Sequential delivery of paclitaxel to colorectal cancer cell by the action of gut microflora enzymes on the degradation of outer chitosan-phytic acid coat and inner porous starch (Reproduced with copyright permission from [99], Elsevier) **(c)** Aptamer CEIMP2 interaction for the enhanced internalization of doxorubicin by HCT116 colorectal cancer cells (Reproduced with copyright permission from [100], Elsevier).

Worldwide, colon carcinoma has been associated with high death counts and mortality rates. Colorectal cancer originates because of the complex extracellular environments in the intestinal lumina. Different changes in the gut microbial flora and the buildup of too many toxins cause cells to keep multiplying [87]. It is one of the most highly malignant tumors and requires a very high dosage of doxorubicin for tumor regression. The dosage of doxorubicin is proportional to the unpredictable side effects of heart and kidney dysfunction. It is necessary to use nanocarriers to lower the dose of doxorubicin, and better permeation and retention are key for targeting and killing tumor cells [93, 94]. Researchers have reported that PEGylated liposomes serve as an efficient method for delivering doxorubicin, thereby increasing the drug's plasma concentration. However, PEG blocks receptor-mediated endocytosis, making it more difficult for the PEGylated liposome to cross the tumor microenvironment, among other effects [50]. On the other hand, the positively charged nanocarrier is an excellent promoter of drug internalization by tumor cells. Askarizadeh and coworkers (2023) have reported the synthesis of cationic liposomes with the MMP-2 (matrix metalloproteinase 2) cleavable PEG-DOPE (1,2-dioleoyl-sn-glycero-3-phosphoethanolamine) linker to not only elevate the half-life of doxorubicin in the blood but also remove the PEG at the site of the tumor microenvironment and expose the cationic liposome for higher drug uptake (Fig. **4a**) [95]. The drug release and pharmacokinetics analysis showed that the PEGylated unsaturated fatty acid chain became less stable when it came to acidity. This led to burst release in the tumor cell's endosome and then in the tumor microenvironment. It contradicted the commercial non-PEGylated liposome, which has saturated fatty acid chains that release doxorubicin at a physiological pH of 7.4. The tested liposomes exhibited varying sizes, zeta potentials, and charges. It worked twice as well on the C26, 4T1, and B16F10 metastatic cancer cell lines (Fig. **4a**) as the DOPE liposome with the positive charge. It's more important that the liposome with PEG-DOPE reduced the amount of doxorubicin that gathered in the heart and kidneys compared to the free drug and the PEGylated liposome [95]. In some cases, other mechanisms have reported active targeting of liposomes. The short peptide A6 from the human urokinase plasminogen activator was found to be able to bind to the membrane receptor CD44 in the C26 colon carcinoma cell. This was superior to targeting the extracellular protein MMP-2. The study demonstrated that the A6 peptide binds to a PEGylated liposome that contains doxorubicin. This facilitated CD44 recognition and expedited doxorubicin delivery. Furthermore, the CD44-A6 interaction, rather than PEG's steric hindrance, governed drug internalization, demonstrating drug uptake without PEG removal [96]. The main problem with the liposome-based system is that it's challenging to get the targeting ligand and PEG to stick together so that the anticancer drug can only reach the tumor. Furthermore, liposomes require stabilizers and demonstrate temperature-sensitive drug internalization [50, 56]. On the contrary, polysaccharide-based systems are easier to develop due to the facile drug encapsulation and active targeting methods. In polysaccharide-based systems, non-covalent interactions regulate the binding and release of active pharmaceutical ingredients, as well as the targeting and uptake of drugs by tumors [17]. It was shown by Solomevich and others in 2023 that a chitosan-dextran phosphate polyelectrolyte hydrogel can interact with ions to deliver doxorubicin and indomethacin under the skin. The use of indomethacin in the hydrogel made doxorubicin more effective at killing cancer cells because it stopped the activity of multidrug resistance protein 1 in those cells. Under normal physiological conditions, the pH-dependent electrostatic interactions between doxorubicin and dextran phosphate carbamate, as well as between indomethacin and chitosan, kept the polyelectrolyte hydrogel stable. The tumor microenvironment, on the other hand, promoted sustained drug release, with a slightly faster release of doxorubicin due to the increased attenuation of electrostatic interactions [97]. Adding zymosan, a β-glucan polymer from Saccharomyces cerevisiae, to doxorubicin caused a similar pattern of drug release that was affected by pH. This is important because it allows for targeted drug delivery. But, when added to C26 colorectal tumor cells as an adjuvant in the form of β-glucan nanoparticles, it lowered the activity of the Wnt/β-catenin pathway and raised the activity of the process that kills cells. By allowing cells to fully absorb the drug, Indomethacin enhanced the cytotoxic activity of doxorubicin. On the other hand, β-glucan worked with the anthracycline drug to kill more tumor cells. However, the cell-killing effects of doxorubicin introduced using polyelectrolyte hydrogel were tested *in vitro*. It was found that positively charged chitosan in the tumor microenvironment was very important for the tumor cells to take up doxorubicin. In contrast, the specificity of β-glucan on tumor cells was not warranted because of a lack of *in vivo* studies [98]. Polysaccharides are inherently hydrophilic; hence, covalent modification is required before introducing hydrophobic drugs [19]. In contrast to liposomes, polysaccharides have a lot of hydroxyl groups. This lets them react in different ways, which makes it possible to create drug delivery systems. Porous starch, created by some enzymes, was joined to octenyl succinic anhydride through esterification. This increased the drug paclitaxel's surface area and ability to encapsulate better (Fig. **4b**) [99]. On the other hand, researchers cross-linked porous starch with hyaluronic acid and further protected it from gastric and intestinal fluid environments through a chitosan-phytic acid envelope to fine-tune the specific and sustained release of paclitaxel in the colon [99]. Peptide-based drug delivery systems have been intriguing because of their flexibility in targeting precise tumor biomarkers [70]. Kianpour *et al*. (2023) reported a very extensive strategy to treat colorectal cancer using aptamers targeted against the overexpressed cell migration-inducing hyaluronidase 2 (CEMIP-2) and identified the conserved pancreatic-derived factor (PANDER)-like sequence consisting of eight glycine as the primary target region in promoting hyaluronic acid degradation and further enhancing cell migration (Fig. **4c**) [100]. *In vitro*, conjugating either a full-length monoclonal antibody or an aptamer (against PANDER) with mesoporous silica not only facilitated the inhibition of anchorage-independent growth of CEIMP2-overexpressed HCT116 cells but also suppressed human umbilical vein endothelial cell (HUVEC) tube formation. Researchers also found that adding doxorubicin to the mesoporous silica killed the cancerous tumor 50% more effectively than using aptamer alone [100]. In 2024, Shen and others made a new peptide-based system by joining the capsid protein of the cowpea melanoma mottled virus to the N-terminal of the elastin-like peptide. This facilitated the encapsulation of the doxorubicin drug. Under physiological pH conditions, the capsid protein's self-assembly into engineered virus-like nanoparticles protected the random release of doxorubicin. Matrix metalloproteases broke down the capsid in the tumor area, leading to the release of doxorubicin (Fig. **4c**). The C26 colorectal tumor very selectively took in doxorubicin, which increased the killing of tumor cells, sped up the maturation of CD4+ T lymphocytes, and increased the number of macrophages. This caused an immune response that greatly reduced the size of the tumor [101]. Iranpour and colleagues (2024) came up with a multimodal therapy that targets the human colon cancer cell HT-29 very specifically. On a zeolitic imidazolate platform, the chemotherapy drug doxorubicin and the radiosensitizer graphene quantum dots were combined. To mitigate hemolysis, the researchers PEGylated the synthesized drug delivery system and derived its high target specificity from the DNA aptamer of the epithelial cell adhesion molecule. The drug carrier was developed by linking the zinc ion with the imidazole, despite the targeting ligand being an aptamer. It was similar to the metal ion-coordinated histidine in the peptide-based nanocarrier system. Histidine is an amino acid with an imidazole ring. In the *in vitro* study, it was intriguing to find that the aptamer-containing drug delivery system made doxorubicin less harmful to CHO cells and more effective at killing HT-29 cells. In line with the research by Shen *et al*. in 2024, the aptamer-conjugated system showed better doxorubicin cytotoxicity in terms of decreasing the size of tumors [102].

### 3.2 Drug Delivery for Breast Cancer Treatment

**Fig. (5):**
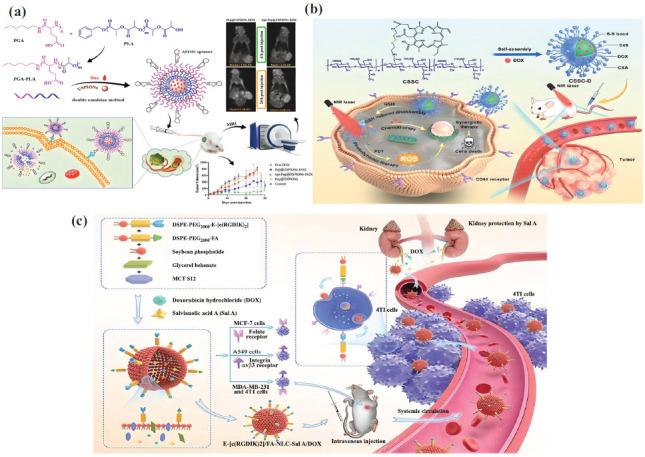
**(a)** Suppression of 4T1 metastatic breast tumor through AS1411 DNA aptamer guided delivery of doxorubicin and ultra-small superparamagnetic iron oxide nanoparticles in PGA-PLA diblock copolymer (Reproduced with copyright permission from [103], Elsevier) **(b)** Chemo-photodynamic therapy inducing cell death of 4T1 breast cancer cell line through co-delivery doxorubicin in chlorin e6 conjugated chondroitin sulphate A nanoparticle (Reproduced with copyright permission from [106], Elsevier) **(c)** Synergistic delivery of doxorubicin and salvianolic acid to MCF-7, A549, 4T1 and MDA-MB-231 breast cancer cell lines through folic acid and RGD peptide functionalized cationic liposomal nanovesicle (Reproduced with copyright permission from [107], BioMed Central journal).

Breast cancer is one of the most prevalent diseases in women, regardless of ethnic and geographic boundaries, and causes high mortality due to its invasiveness and multidrug resistance [88]. Researchers attribute the widespread prevalence of breast cancer to the unprotected anatomical features of breast epithelial cells and the ongoing alterations in cell physiology caused by hormonal fluctuations during menstrual and fertilization events. As compared to other tumors, breast cancer is associated with highly complex stroma and thick blood vasculature, promoting extremely high malignancy and early metastasis [89]. Furthermore, the probability of metastasis is substantial based on the reported literature on breast carcinoma. It forms a significant setback in the development of anti-tumor drugs at the advanced stages of tumor progression [92]. Chemotherapy is the most commonly used treatment to control the severity of tumor metastasis and decrease the rate of tumor growth among existing treatments. As discussed in the previous sections, chemotherapy requires extreme target specificity to mitigate or nullify systemic toxicity [5]. Researchers mainly developed natural polymer-based nanocarriers to confine the distribution of chemotherapeutic drugs in the tumor's microenvironment and reduce the sensitivity of normal cells to the drug's cytotoxicity [13]. Hasannia *et al*. (2023) reported the synthesis of peptosomes (similar to the structure of a liposome), consisting of poly (L-glutamic acid) and polylactic acid, to encapsulate the hydrophilic doxorubicin in the aqueous core and the hydrophobic ultra-small superparamagnetic iron oxide nanoparticles in the bilayer (Fig. **5a**) [103]. Additionally, they tagged the peptosome with a DNA aptamer to specifically narrow its interaction with surface biomarkers on murine mammary carcinoma (4T1) cell lines. The evaluation of drug release in phosphate-buffered saline and citrate buffers, which indicate the pH values of blood plasma and tumor microenvironment, respectively, revealed a gradual release of doxorubicin over a period of 240 hours. However, the cumulative drug release was less than 15% in phosphate-buffered saline. Meanwhile, there was a sustained release of 25% of doxorubicin in the citrate buffer. Even though the conjugation of aptamer in peptosome showed no significant difference in the final cellular cytotoxicity at the doxorubicin concentration of 20 µg/mL, there was a remarkable reduction in the tumor volume of 4T1 cell lines *in vivo* (Fig. **5a**). Furthermore, the quantification of doxorubicin through fluorescence intensity-guided flow cytometry revealed a dramatic reduction in drug accumulation in the heart, thereby alleviating doxorubicin's systemic toxicity. On the contrary, magnetic resonance imaging warranted enhanced accumulation of doxorubicin in the tumors, and the DNA aptamer in the peptosome significantly extended the tumor's residence time [103]. In addition to aptamers, studies have shown that integrating short peptide recognition sequences into solid lipid nanoparticles (SLN) broadens the pharmacokinetics of doxorubicin in breast cancer treatment, both *in vitro* and *in vivo*. The selection of the RGD (arginine-glycine-aspartate) tripeptide sequence was based on the profound overexpression of αvβ3 integrins in the MCF-7 breast cancer cell line, and the research group evaluated the drug delivery to a doxorubicin-resistant cell line named MCF-7/ADR cells. The variable cross-linking density between glycerine monostearate and adipic acid dihydrazide, which forms the SLN, facilitated drug release in a pH-responsive manner. The presence of RGD in SLN augmented the important pharmacokinetic factor, namely the area under the plasma concentration-time curve, by fivefold, and the significant finding was the accumulation of doxorubicin in the multidrug-resistant cell line to exhibit cytotoxicity above the threshold concentration of 39 L/kg/h. Notably, the free drug doxorubicin had very little effect on the MCF-7/ADR cells, with a half-maximal inhibitory concentration of 47 µM. However, the release of doxorubicin using the RGD-modified SLN improved its activity against drug-resistant cells, and the half-maximal inhibitory concentration was reduced to 4.1 µM. As a result of an improvement in the inhibitory action of doxorubicin against the highly drug-resistant breast cancer cell line, the tumor volume decreased from 2258 cubic millimeters to 405 cubic millimeters [104]. Mutations in three highly deregulated proteins, namely the estrogen receptor (ER), progesterone receptor (PR), and human epidermal growth factor 2 (HER2), determine the grade of breast cancer. Benign tumors have a single mutation, and the progression to a highly invasive malignant stage occurs as a result of simultaneous mutations in three proteins. Reports have shown mutations occur in one protein due to a single amino acid substitution and loss of function in another [89, 92]. PEGylated liposome carrying doxorubicin has been the most commonly recommended drug delivery system for the treatment of breast cancer [50]. However, studies have highlighted the requirements of synergistic chemotherapeutic drug actions to maximize tumor regression [4]. Ghosh and colleagues (2021) formulated a pegylated liposomal nanocarrier by adding alkaloid vincristine sulfate to doxorubicin and demonstrated the synergistic chemotherapy effect on reducing the severity of triple-negative metastatic breast cancer. More importantly, the combined drug action inhibited drug efflux by selective ammonium ion gradient in tumors and promoted higher tumor internalization. The results from cell viability, flow cytometry, and confocal microscopy showed that combined chemotherapy not only increased the amount of doxorubicin inside cells by a factor of several, but it also helped stop the cell cycle at the G2/M transition by increasing apoptosis [105]. In the case of polysaccharide-based nanocarriers, negatively charged polysaccharides with negative zeta potential are considered for intravenous administration of chemotherapeutic drugs [19]. First and foremost, the breast carcinoma, unlike other cancer cells, shows specific internalization of polyanionic nanocarriers due to the exuberant expression of CD44 on triple-negative highly metastatic carcinoma [89]. Chondroitin sulfate, a polyanionic glycosaminoglycan, undergoes receptor-mediated endocytosis through CD44 interaction. Furthermore, it is a substitute for hyaluronic acid because of its reduced susceptibility to degradation by glycosyl hydrolases present in metastatic tumor stroma. But chondroitin sulfate reacts more with plasma proteins (platelets) because its zeta potential value is higher than that of hyaluronic acid. As a result, the design of a chondroitin sulfate-based nanocarrier necessitates surface charge optimization to prevent uncontrolled drug release due to native polymer cross-reactivity [106]. Yu *et al*. (2024) demonstrated a highly controlled drug release to the 4T1 cells (triple negative breast cancer cell line) in BALB/c mice bearing tumor xenograft by altering the surface charge of chondroitin sulfate. The disulfide linkage between chondroitin sulfate A and photosensitizer chlorin e6 produced an amphiphilic polymerosome by self-assembly with non-covalently bound doxorubicin in the central hydrophobic region (Fig. **5b**) [106]. On the outer surface, the negatively charged group of polysaccharides was visible. The study found that photodynamic therapy fine-tuned intracellular delivery of doxorubicin, as well as the generation of free radical species and singlet oxygen. Moreover, irradiation of a laser at 660 nm elevated the synergistic action of doxorubicin and free radical species on tumor cell death [106]. Co-delivery of two chemotherapeutic drugs using folate and RGD peptide-functionalized cationic liposomes reduced the metastasis of the most commonly reported MCF-7, A549, 4T1, and MDA-MB-31 breast cancer cell lines (Fig. **5c**) [107].

### 3.3 Multifaceted Drug Delivery Through Integration of Natural Polymers

**Fig. (6):**
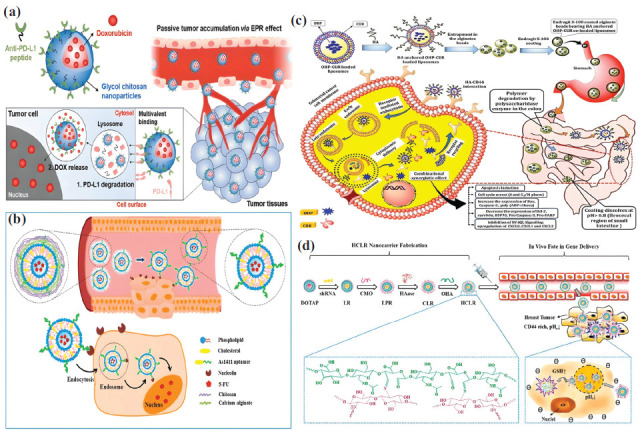
**(a)** Synergistic delivery of doxorubicin and anti-PD-L1 peptide in the treatment of CT26 colon tumor bearing mice using all-in-one chitosan glycol nanoparticles (Reproduced with copyright permission from [112], Elsevier) **(b)** Colon specific delivery of 5-fluorouracil through chitosan-alginate protected aptamer functionalized liposomal nanocarrier (Reproduced with copyright permission from [113], Elsevier) **(c)** Administration of alginate encapsulated HA conjugated liposomal nanoparticle for the combined delivery of oxaliplatin and curcumin to oxaliplatin resistant HT-29 colorectal cancer cells (Reproduced with copyright permission from [118], Elsevier) **(d)** Stepwise synthesis of HCLR (HA/HAase/CS/liposome/survivin-shRNA) drug delivery system and stimuli responsive delivery of sh-RNA to MDA-MB-231 breast tumor (Reproduced with copyright permission from [125], American Chemical Society).

The grade of malignant tumor increases the complexity of treatment procedures. During the transition of hyperplastic tumor growth to invasive neoplasm, reports have shown the existence of at least three grades [108]. One treatment method called monotherapy increased survival rates for grade III because the target mutant protein was harmless. This could be an oncoprotein that is overexpressed or a critical tumor suppressor protein that is downregulated [109]. However, high-grade tumors carry multiple mutations, and so the development of drug delivery systems becomes cumbersome [92]. It has been shown that all anti-tumor metabolites, antibodies, and alkylating agents can work together in one delivery system. However, the conjugation of multiple drugs poses steric hindrance and introduces limitations in the stimuli-responsive drug release [110]. Conversely, the combination of natural polymers addresses the invasive nature of metastatic tumors by enhancing the cytotoxic effects of administered anti-cancer drugs (Table **2**) on both the tumor and surrounding stromal cells [111]. Song *et al*. (2023) reported delivery of doxorubicin in amphiphilic chitosan glycol nanoparticles through intravenous administration in CT-26 colon-tumor-bearing mice (Fig. **6a**) [112]. The hydrophilic chitosan underwent a 1:100 cross-linking with cholanic acid to encapsulate the doxorubicin, and a click chemistry reaction covalently attached its free amine groups to the anti-PD-L1 peptide. Apart from the drug administration, the study showed that the covalent linkage of anti-PD-L1 peptide to chitosan glycol nanoparticle promoted multivalent interactions with the tumor surface protein PD-L1 (programmed death ligand 1) and enhanced its lysosomal degradation (Fig. **6a**). As a result of PD-L1 downregulation, there was additional tumor lysis by the cytotoxic T lymphocytes. Furthermore, the study demonstrated 60% regression in tumor metastasis by the combination of chemotherapy and immunotherapy in a single delivery system [112]. Polyelectrolytes have been produced through ionic interactions between two oppositely charged polysaccharides, and these show insignificant alterations in molecular structures with changes in pH [19]. To protect 5-flurouracil from the pH variations in the stomach and small intestine, it was administered in a liposomal vehicle surrounded by the chitosan-alginate polyelectrolyte complex (Fig. **6b**) [113]. The enzymatic hydrolysis of polysaccharides facilitated the delivery of HT-29 colorectal cancer. Furthermore, the conjugation of a single-stranded DNA aptamer, which targeted overexpressed nucleolin in the cytoplasm of HT-29, significantly reduced the half-maximal inhibitory concentration of 5-flurouracil [113]. Delivering drugs to the colon necessitates a release profile that contradicts the tumor microenvironment. In other words, orally administered drugs undergo target-specific release with an increase in pH and show extremely small release in an acidic environment (*e.g*., gastric fluid) [114]. Most studies on the development of nanocarriers as anti-cancer drug delivery systems generally report charge-independent variations in the particle size, which BET and zeta potential analyses have supported [81, 115, 116]. Even though the diameter of the liposome attached to polysaccharides/polyelectrolytes and aptamers changed, the zeta potential value didn't change much [113]. In 2023, Pai and Jin looked at how two different negatively charged polymers—γ-polyglutamic acid and fucoidan (sulfated polysaccharide)—affected the release of irinotecan, a drug that blocks topoisomerase I, in the HCT116 colorectal cancer cell line. When fucoidan was mixed with chitosan in a polyelectrolyte complex, it showed a lot of antitumor activity. Factors promoting elevated tumor apoptosis in the case of an irinotecan-loaded fucoidan-based nanocarrier were the secondary interaction of fucoidan with p-selectin (a biomarker in HCT116), lower half-maximal inhibitory concentration, and involvement of fucoidan in the activation of apoptotic proteins, viz., caspase 9, caspase 3, and poly (ADP ribose) polymerase. However, the fucoidan-based system was not specific to HCT116, and it caused a significant decrease in cell viability of the L929 cell line compared to the γ-polyglutamic acid-based delivery system [117]. Tiwari *et al*. (2022) created a cascade drug delivery system using polysaccharides, liposomes, and a chemotherapeutic drug to treat HT-29 colon cancer cells that are resistant to oxaliplatin in living organisms (Fig. **6c**) [118]. A study demonstrated the efficacy of curcumin in mitigating the drug resistance of HT-29 by inhibiting the NF-κB pathway in B cells, preventing tumor progression. So, the delivery system had a liposome that had both oxaliplatin, which is hydrophilic, and curcumin, which is oleophilic (Fig. **6c**). Additionally, the covalent attachment of hyaluronic acid to the liposome through carbodiimide chemistry facilitated the liposome's active targeting of the CD44-expressing HT-29. Considering the oral route of administration, the alginate polysaccharide was used as the outermost shell to minimize drug release from the liposome in the acidic environments of the stomach and small intestine. In summary, the hydrolases produced by the gut microflora in the colon stimulated the release of drugs. The receptor-mediated endocytosis of the liposome through hyaluronic acid-CD44 interaction followed this process (Fig. **6c**). In the last step, lysosomes were integrated, oxaliplatin and curcumin were released inside cells, and the combined therapeutic effect led to the death of tumor cells [118]. The route of administration for breast cancer treatment has been intravenous injection in most cases [119]. Hence, the delivery system includes hyaluronic acid-modified liposomal nanocarriers to prevent drug clearance by reticuloendothelial mechanisms. Many studies have demonstrated the importance of hyaluronic acid in increasing drug plasma concentration and bioavailability and targeting the most commonly expressed CD44 surface protein in all types of breast carcinomas [120]. At different stages of tumor growth, the environment around the tumor is always changing. Most metastatic breast carcinomas are surrounded by a thick stroma made up of extracellular matrix components like collagen, hyaluronic acid, fibroin, and others [89, 92]. The type of collagen expressed in the tumor stroma, particularly collagen type IV, differs widely from other normal tissues. This unique expression pattern provides a suitable target for designing nanocarrier-based drug delivery systems, as it allows for specific and effective drug delivery to the tumor site. Reports have shown the predominant expression of collagen type IV in the stroma of triple-negative breast cancer [121, 122]. Ikeda-Imafuku *et al*. (2022) developed hyaluronic acid-conjugated peptides and sulfhydryl proteases (bromelain) using the N-hydroxy succinimide/1-ethyl-3-diaminopropyl carbodiimide chemistry. *In vivo*, they identified the anti-C4BP antibody binding with collagen type IV (C4BP) as the best nanocarrier for exceptional tumor accumulation in the 4T1 cells bearing xenograft mice. HA also improved the drug's biodistribution by raising the plasma concentration. The peptide, on the other hand, directed the protease action on collagen type IV in the tumor stroma, breaking down the extracellular matrix. As a result, the system significantly facilitated liposomal doxorubicin tumor internalization [123]. Breast cancer is versatile, with multiple mechanisms of origin and progression. Hence, identifying breast cancer types and analyzing the tumor microenvironment differs from one case to another, making the treatment more personalized and complicated [124]. The development of one ideal nanocarrier-based delivery system to regress predominant types of breast carcinomas is rare [88]. Chen and his colleagues did a study in 2020 that used all three types of natural polymers—polysaccharide-based, liposome-based, and peptide-based nanocarriers—together in one system to deliver RNAi (short hairpin RNA against surviving protein) to two types of very aggressive breast tumors (MCF-7 and MDA-MB-231) (Fig. **6d**) [125]. To create the drug delivery system, they first incorporated RNAi into the cationic DOTAP liposome. Chitosan was then functionalized with 2-mercaptoethyl oleate through disulfide linkage. To make the drug delivery system, modified chitosan oleate was added, and hyaluronidase was electrostatically bound to the cationic liposome in the inner core (Fig. **6d**). On the other hand, the system's outer shell included the covalent cross-linking of oxidized hyaluronic acid with chitosan. In the tumor microenvironment, this complex drug delivery system demonstrated stimuli-responsive release based on acidity and GSH levels. A decrease in pH hydrolyzed the outer shell, promoting the release of hyaluronidase to degrade the tumor stroma and guide the drug's internalization through CD44-hyaluronic acid interaction. Furthermore, the intracellular glutathione levels, which favor redox biochemical reactions, reduced the disulfide bond in modified chitosan oleate, stimulating the release of RNAi (Fig. **6d**). Flow cytometry, fluorescence resonance energy transfer, and confocal microscopy all showed that the MDA-MB-231 cell line took in more than 85% of the drug. Even though only 70% of the drug was taken up by MCF-7, the all-in-one drug delivery system that combined active and passive targeting helped two different types of breast carcinomas shrink [125].

## 4 CLINICAL STATUS OF THE NATURAL POLYMER DRUG DELIVERY SYSTEMS

**Fig. (7):**
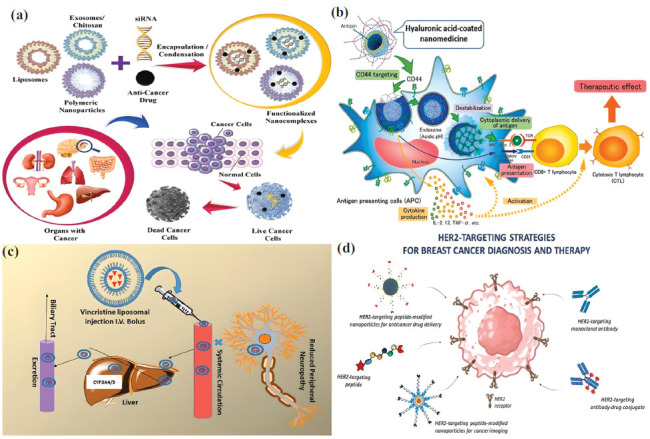
**(a)** Multiple drug delivery systems carrying anticancer drug and siRNA for demonstration of chemotherapy in clinical trials (Reproduced with copyright permission from [137], Elsevier) **(b)** Principal mechanism of drug internalization and antitumor response using hyaluronic acid based nano delivery system (Reproduced with copyright permission from [139], American Chemical Society) **(c)** Minimal first pass metabolism and diminished peripheral neuropathy of clinically approved vincristine loaded liposomal formulation (Reproduced with copyright permission from [142], Springer) **(d)** Monoclonal antibody-based treatments targeted against HER-2 positive breast carcinoma in phase III trials (Reproduced with copyright permission from [146], MDPI journal).

After explaining the promising outcomes of the multifaceted drug delivery system, it represents an absolutely safe and extremely effective method for administering chemotherapeutic drugs. However, the fabrication and evaluation of complex natural polymer conjugated systems have been majorly restricted either to *in vitro* cell culture studies or *in vivo* studies in tumor-grafted mice [126]. Several research reports combine natural polymers to enhance therapeutic potential, sharpen tumor specificity, promote tumor accumulation, and minimize drug efflux [124, 127, 128]. Furthermore, numerous studies have demonstrated how physical stimuli can enhance the uptake of chemotherapeutic drugs by tumor cells [106, 129]. The question is whether these polymers, in their widely varied structures, pose toxicity to human subjects or if there is a lack of successful validation studies on clinical trials. The main challenge is the former, due to the unpredictable reactions of the complex delivery system with the host immune system and the less-known detoxification mechanism of the modified forms of natural polymers into harmless cellular metabolites [130]. In clinical trials, there aren't as many ways to give the drug as there are in pre-clinical studies using animal models. This is because there are more likely to be multiple non-specific interactions between the drug delivery system and the host cells that are responsible for the main drug administration routes [131]. For example, oral administration of a drug requires consideration of pH oscillations in the gastrointestinal compartment, action of digestive enzymes (proteases) in the gastric juices, and diffusion barriers across tight epithelial junctions to retain the bioavailability and minimum inhibitory concentrations for efficient cancer chemotherapy [132]. Giving chemotherapy agents through an intravenous route still carries the risk of systemic toxicity, adverse inflammatory reactions (stimulation of interleukin and cytokine secretions), lymph node infiltration, and uneven distribution in the primary metastatic tumor site [133]. Clinical trials that employ multiple drug delivery systems often face a significant issue: the treatment's short-term effectiveness is short-lived, as the reversal of antiproliferative conditions allows the tumor to grow and cells to move again. Moreover, tumor cells can regain multidrug resistance, even after a brief relapse [131]. In clinical trials, the desired outcome of cancer chemotherapy is to increase the survival rate and extend the patients' lifespan rather than complete elimination of the metastasizing tumor. Because of this, most people agree that using a single natural polymer-based delivery system is the safest and most gentle way to give drugs at different stages of clinical trials [134]. Despite their lower yields and risk of viral infection/cross-contamination, natural polymers possess bioactive properties that make them suitable for drug delivery applications [135]. Researchers have evaluated polysaccharide-based nanocarriers, composed either of chitosan, cyclodextrin, or hyaluronic acid (Table **3**), in the treatment of colorectal and breast carcinomas and have intensely investigated each individual formulation in phase II and phase III trials [129]. As a positively charged material that can selectively deliver drugs to human cells (as opposed to animal cells or bacterial cells) and has excellent biodegradability, chitosan is seen as one of the best renewable polymers (Fig. **7a**) for making doxorubicin and paclitaxel-enclosed delivery systems [20, 136, 137]. Cyclodextrin, known for its dual lipophilic and hydrophilic nature, adsorbs hydrophobic drugs in the interior and protects them from immune cells and antibodies in the circulatory system without the need for additional chemical conjugation [138]. Because of its widespread presence in body fluids, hyaluronic acid is the least immunogenic polysaccharide (Fig. **7b**) [139], and its extremely specific interaction with the CD44 receptor on tumor cells makes it the most attractive delivery system for tumor regression [140]. In the case of liposomes, attaching polyethylene glycol is the safest cargo molecule for targeted delivery of irinotecan and 5-fluorouracil to pancreatic tumors. Moreover, PEGylated liposomes conjugate with FDA-approved monoclonal antibodies (trastuzumab, pembrolizumab) to elicit combined actions of chemotherapy and immunotherapy in ovarian and breast cancers [141]. A chemotherapeutic drug and a potent microtubule formation inhibitor named vincristine (Fig. **7c**) have been conjugated into sphingomyelin/cholesterol liposomes (Table **3**) to generate a clinically safe liposomal delivery system (Marqibo) for treating acute lymphoblastic leukemia [142]. Liposomes loaded with siRNA called Atu027 have been approved for phase II clinical trials. This siRNA can reduce the expression of protein kinase 3 and stop pancreatic tumor metastasis. Different clinical trials have deeply investigated photodynamic therapy using liposomes, with a prominent example being the dual irinotecan and cetuximab-loaded liposome-carrying photosensitizer (benzoporphyrin derivative) that mitigates desmoplasia in pancreatic cancer [141]. The transition of liposomes from simple PEG-conjugated (first generation) to antibody-conjugated (second generation) and cell membrane-coated systems (third generation) improved biodistribution and tumor accumulation, as well as promoted sustained drug action on cumulative tumor regression [143]. Proteins, instead of artificially synthesized peptides, coming from natural sources such as silk fibroin, collagen, and gelatin have broad applications in drug delivery. For instance, red blood cell membrane-coated gelatin had pharmacological activity equivalent to the antibiotic vancomycin in the MRSA-infected mouse models [135]. Exquisite preclinical tests that reported hyaluronidase (which breaks down the complicated tumor stroma) for tumor targeting are no longer used in clinical settings because of thrombosis formation [144]. Furthermore, peptide-based nanocarriers are exceptionally versatile in terms of nanostructures and oncoprotein binding sequences, allowing for rapid *in vitro* internalization of chemotherapeutic drugs [70]. However, the undeniable toxicity of synthetic peptides on stromal cells in general and extracellular MMPs in particular prevents their acceptance for advanced clinical trials [130]. Peptide-based nanocarriers have been found to be unsuitable in phase III clinical trials due to the diverse antibody and humoral responses that exist in the body and vary from one patient to another, according to detailed reviews [145]. The most specific and extremely target-sensitive peptide-based systems recommended by the FDA for phase III clinical trials are monoclonal antibodies (Table **3**). Moreover, rational engineering of the developed antibodies' structures, particularly the carboxyl-terminal or Fc regions, has produced several potent drugs against triple-negative breast carcinoma and glioblastomas (Fig. **7d**). A notable example is the chimeric anti-HER2 monoclonal antibody consisting of Fc domain-tailored trastuzumab [146]. A novel and precise delivery of drugs has been carried out through microneedle technology involving continuous infusion of active agents through microscopic pathways across the *stratum corneum*. These have been especially useful in the delivery of immunomodulators as well as antibodies in skin cancer treatment [147].

## 5 DISCUSSION

Conventional chemotherapeutic treatments for tumor regression have limited survival rates due to several drawbacks, such as attenuated pharmacokinetics, systemic toxicity, insignificant tumor accumulation, and drug uptake [148]. The primary restriction on chemotherapy efficacy is multidrug resistance, as it causes frequent recurrence of the disease in the majority of malignant tumors [149]. Considering the shortcomings of monotherapy, the treatment approaches are customized by combining chemotherapy with radiotherapy, immunotherapy, photodynamic therapy, *etc*. [4]. Even though combinatorial therapy increases the survival rate of patients, it is associated with a long-term risk of health complications [150]. As a result, it is inevitable that a single, consolidated therapy will be created that has much higher antitumor potential, cytocompatibility, and much lower tumor rejection [151]. The drug delivery system's design considers passive targeting through the larger tumor vasculature, necessitating the development of a nanocarrier-based system [19]. However, passive targeting through EPR is not sufficient for tumor internalization due to the thick stroma, dense extracellular matrix, and the tumor's inherent drug resistance. Tumors have variable extracellular environments depending on the stage of tumor progression. Most invasive metastatic tumors have complex stroma to hinder the transportation of nanocarrier-based drug delivery systems from the endothelial gap junction to the tumor cell surface. Thus, the incorporation of multiple functional components is critical to achieving the true intended potential of chemotherapeutic drugs [152]. Considering the magnitude of tumor growth and metastasis, the combination of passive and active targeting approaches provides a consolidated treatment method with the advantages of elevated recovery rate, diminished systemic toxicity, and improved attenuation of tumor volume [153]. Active targeting using polysaccharide-based nanocarriers requires modification. In addition, the guided tumor internalization property comes from the preferential incorporation of negatively charged hyaluronic acid into neutral polysaccharides through chemical conjugation. The CD44 receptor, a widely expressed cell surface protein in most tumor cells, controls this navigation mechanism through receptor-mediated endocytosis [43]. On the other hand, researchers have blended polysaccharide-based nanocarriers into polyelectrolyte complexes to encapsulate hydrophobic drugs and minimize drug clearance by the reticuloendothelial system and acidic pH environments. Moreover, polyelectrolytes have been integrated with liposomes to carry multiple chemotherapeutic drugs [113, 114]. Liposomes, though associated with lower immunogenicity and longer circulation time, are leaky and release the encapsulated drug in the surrounding environments to cause a substantial drop in tumor accumulation [50]. Liposomes, in contrast to polysaccharides, can transport a variety of hydrophobic, hydrophilic, and amphiphilic drugs effectively. The size of the liposome varies depending on the endothelial gap junction in the tumor vasculature. However, liposomes have limitations in crossing the tumor microenvironment [51, 53]. Researchers have found that attaching positively charged chitosan to phospholipids through disulfide and carbodiimide linkages makes the liposome more stable and makes it easier to add extra therapeutic enhancers like proteases, hyaluronidases, iRGD (cyclic arginine-glycine-aspartic acid) sequences, non-covalently linked hyaluronic acid, and more [154–156]. Peptide-based carriers are highly specific and carry out the dual function of active targeting and tumor suppression [70]. The synthesis of hydrophobic amino acids and subsequent attachment with polar amino acids produce a peptosome, which forms a self-assembled structure to encapsulate hydrophobic chemotherapeutic drugs [157]. Designing sequences that bind to tumor-specific extracellular proteins provides a valuable targeting system to identify particular tumor tissue types. Researchers have studied the extent of metastasis using a fluorescently labeled peptide-based nanocarrier system [77]. Peptides self-assemble into nanorods, nanosheets, and micelles depending on pH and regulate drug release according to the three-dimensional structure. For instance, few cyclic peptides exist as nanospheres under physiological pH and become elongated and ruptured in acidic tumor microenvironments, promoting the dissociation of the non-covalently linked doxorubicin into tumor cells [81, 158]. Peptides, in contrast to polysaccharides and liposomes, have restricted pharmacokinetics due to their interactions with various plasma proteins and host immune systems, resulting in protease-mediated degradation and limited tumor accumulation [159]. Oral administration of chemotherapeutic agents using polyelectrolyte-based liposomal systems has proven successful in treating colon carcinoma, primarily due to enhanced permeation and retention effects. Polyelectrolytes also keep hydrophobic drugs safe from the acidic fluids in the stomach and intestines [113, 114, 116]. In contrast, the tumor environment determines targeted drug release in breast cancer scenarios, and more specifically, the treatments reported for chemotherapy against invasive carcinomas such as MCF-7 and 4T1 have been through multiple adjuvant-based nanocarrier systems (active targeting) [103-106]. In order to reduce the administered dose equivalent of chemotherapeutic drugs and customize the intensive targeting against metastatic tumors, researchers have combined natural polymers into a large, layer-by-layer assembled cascade delivery system [125].

## CONCLUSION

The multifaceted nano-delivery systems are associated with extremely high tumor internalization and substantial tumor volume attenuation. From the point of view of how drugs interact with tumor cells, having multiple ligands is necessary to increase the concentration of chemotherapeutic drugs inside cells and decrease the ability of metastatic tumors to spread. More importantly, the multifaceted nanocarrier-based system made of natural polymers not only eliminates the need for radiotherapy and immunotherapy, but also lowers the risks of immunogenicity, non-specific cytotoxicity, tumor rejection, and other issues [160, 161].

## Figures and Tables

**Table 1:** Properties and pharmacokinetics of polysaccharide-, lipid-, and peptide-based nanocarriers in cancer chemotherapy.

**Polymer** **Composition**	**Preparation Method**	**Physical** **Property**	**Drug**	**Targeting Mechanism**	**Pharmacokinetic Parameters**	**Tumor Action**	**References**
Pullan, hyaluronic acid	Acid cleavable pullalan-doxorubicin was synthesized by carbodiimide chemistry and thiolated hyaluronic acid was added by click chemistry	Mean particle size: 68.47 nm. Zeta potential: -32 mV	Encapsulation of Lapatinib in polysaccharide-doxorubicin conjugate	Passive targeting through EPR effect and active targeting through CD44 receptor internalization	Tumor growth inhibition of 93% at a dosage of 1 mg/kg. Cumulative release and IC_50_ of doxorubicin were 80% and 0.98 µg/mL. Cumulative release and IC_50_ of lapatinib were 60% and 6.06 µg/mL	Reduced expression of P-glycoprotein (P-gp) in multidrug resistant MCF-7 cell line (breast cancer). Tumor weight reduction by 80%	[44]
Amphiphilic phosphonium chitosan nanomicelle	Room temperature synthesis of N-acetyl-L-phenylalanine-(4-carboxybutyl) triphenylphosphonium bromide chitosan by carbodiimide chemistry	Average particle size: 103 nm. Zeta potential: 32.2 mV	Zinc phthalocyanine was encapsulated at critical micelle concentration of 5 mg/L through ultrasonic self-assembly	*In vitro* Nanomicelle internalization by lattice-mediated endocytosis (active targeting)	Encapsulation efficiency of 89.4%. Photodynamic therapeutic release using laser radiation (660 nm, 0.5 W/cm^2^) was 64.35% at pH of 6.5	Upregulation in caspase 3 apoptotic pathway by 11.4% in human pancreatic cancer cells (Panc-1 cells)	[47]
PEGylated liposome, octreotide peptide	Conjugation of octreotide with cationic PEGylated DSPE in phosphate buffered saline by carbodiimide chemistry	Mean size: 152 nm. Zeta potential: 4.10 mV	Encapsulation of lipocalin 2 (Lcn2) small interfering RNA (Lcn2 siRNA)	Active targeting through somatotropin receptor mediated endocytosis.	Encapsulation efficiency of 69.5%. Cumulative release of 80% drug using octreotide peptide decorated liposome at pH 6.8. Similar drug release using PEGylated liposome with fetal bovine serum (FBS) supplementation.	Downregulation of lcn-2 expression in triple-negative breast cancer cell line MDA-MB-231 (cell viability = 20%). Suppression of angiogenic factors (VEGF-A concentration < 200 pg/µg)	[58]
PEGylated liposome (sphingomyelin and DSG) and anti-PD-L1 peptide	Micelle transfer method for insertion of target peptide into drug loaded liposome	Particle size: 113.6 nm. PDI: 0.038	Direct loading of docetaxel into liposome at elevated temperature	EphA2-targeted liposomal taxane (active targeting)	Drug to lipid ratio = 373.1 g/mol. Increase in AUC of docetaxel from 52.1 to 785 ng/mL/hour. Dosage of drug for tumor volume reduction from 1,500 to 500 mm^3^ was 59 mg/kg in EMT-6 tumor	Regression of non-small cell lung carcinoma and triple negative breast cancer by 60% in the presence of cytotoxic T lymphocytes	[65]
Tyrosine-tryptophan dipeptide-based nanoparticles	Dipeptide self-assembled into nanorods and nanospheres through hydrogen bonding, Π-Π stacking and Zn (II) coordination	Average diameter of self-assembled 3D spheres: 1794 nm	Encapsulation of doxorubicin during the zinc ion mediated self-assembly of dipeptides	Aptamer directed against epithelial cell surface marker (EPCAM) facilitated chaperone mediated endocytosis (active targeting)	Drug loading capacity = 16.6%. Intracellular drug release reached 60% after 30 h. Structural rigidification by zinc ion shifted fluorescence from ultraviolet to visible region.	Cell viability reduction by 50% in lung cancer epithelial cell line (A549). Lowest accumulation of doxorubicin in cardiomyocyte.	[77]
RGD peptide spherical nanoparticles (LKR)	Solid phase peptide synthesis (with acetylation of N-terminus and amidation of C-terminus) followed by self-assembly in HEPES buffer (pH = 7.4)	Particle sizes of nanostructures at pH 7.4 and 6.0 were 50-60 nm and 220-460 nm respectively. Zeta potential = 20-25 mV	Ultrasound assisted entrapment of doxorubicin in RGD nanoparticles	Enhanced permeation and retention effect coupled with active targeting through αυβ3 integrin	pH responsive release of drug. Cumulative release <20% at pH 7.4 and burst release of doxorubicin up to 80% at pH 6.0.	Anti-tumor efficiency of doxorubicin loaded nanoparticle against H22-hepatoma bearing BALB/c mice was 77%	[81]

**Table 2:** Composition of multifaceted nanocarriers along with pharmacodynamics of drug delivery for treatment of colorectal and breast cancers.

**Nanocarrier Composition**			**Morphology and** **Preparation**	**Therapeutic Agents**		**Pharmacodynamics**	**Key Finding**	**References**
**Polysaccharide**	**Lipid**	**Peptide/** **Aptamer**		**Primary Drug**	**Secondary Drug**			
Glycol chitosan	5β- Cholanic acid (sterol)	Anti-PD-L1 peptide	Conjugation of peptide with nanoparticle by copper free click chemistry reaction. Mean diameter of the spherical nanoparticle = 220-230 nm	Doxorubicin	Anti-PD-L1 antibody	Intravenous administration and drug delivery through both passive and active targeting routes. During active targeting, multivalent binding of nanoparticle to PD-L1 induced its lysosomal degradation.	All-in-one glycol chitosan nanoparticles prevented PD-L1 (programmed death ligand-1) recycling and promoted immune checkpoint blockade. Delivery of doxorubicin strengthened immunogenic cell death of CT-26 colorectal cancer cells and caused tumor regression by 60%.	[112]
Chitosan-alginate	Diastearoyl phosphatidyl ethanolamine-methoxy polyethylene glycol	AS1411 aptamer	Spherical nanoparticles with mean diameters of 120 nm and 170 nm. Concentration of loaded 5-fluorouracil = 20 mM	5-Fluorouracil	Anti-nucleolin aptamer	Attenuated degradation of nanoparticles in gastric and intestinal fluids.	Aptamer-conjugated liposome induced increased cell death of HT-29 tumor cells. Cell viability < 60% .	[113]
Chitosan (CS), fucoidan (FCD)	No lipid	γ-polyglutamic acid (γ-PGA)	Size of γ-PGA nanoparticle = 146 nm. Size of FCD nanoparticle = 230.8 nm. PDI < 0.3. Zeta potential = 25-27 mV	Irinotecan	Fucoidan	Specific interaction of fucoidan with p selectin surface protein. IC_50_ of FCD nanoparticle was 2.4 times lower than γ-PGA nanoparticle. Drug release was 80% in 96 h at pH 7.4.	Upregulation of apoptosis in HCT-116 colorectal cancer cell line through 2.3-, 3.5- and 6.3-fold increases in the expression of caspase 3, caspase 9 and poly (ADP ribose) polymerase.	[117]
Hyaluronic acid, Sodium alginate	Distearoylphosphatidylethanolamine and hydrogenated soy phosphatidyl choline	No peptide/aptamer	Size of liposomal vesicle = 113 nm. Entrapment efficiency of oxaliplatin = 30%. Entrapment efficiency of curcumin = 73%. PDI > 0.5. Zeta potential = -9.25 mV	Oxaliplatin (OHP)	Curcumin (CUR)	Hyaluronic acid coating enhanced the cytotoxicity of the liposomal delivery system by nearly three-fold. IC_50_ of HA conjugated liposome (loaded with both drugs) was 1.9 µM.	Dramatic reduction in cell viability from 30% (only OHP loaded liposome) to 7.2% (OHP+CUR loaded liposome) OHP resistant HT-29 cell line	[118]
Hyaluronic acid	Liposomal doxorubicin	Collagen type IV binding peptide (C4BP)	NA	Bromelain	Doxorubicin	Intravenous administration of HA-C4BP conjugate along with liposomal doxorubicin containing 5 mg/kg bromelain and 3 mg/kg doxorubicin.	Decrease in tumor volume of triple negative breast cancer 4T1 from 1000 mm^3^ to 200 mm^3^. No survival and 100% survival for the respective control and treated 4T1 bearing mice after 30 days.	[123]
Amphiphilic chitosan, hyaluronic acid	DOTAP, methyl oleate	Hyaluronidase	Hydration size = 105 nm, PDI = 0.118. Change in zeta potential from -23.1 mV to 29.9 mV with pH drop from 7.4 to 6.5	Survivin small hairpin RNA	NA	Passive delivery in acidic tumor microenvironment and active targeting through CD44 receptor. High intracellular glutathione concentration (10 mM) promoted release of sh-RNA. More than 85% drug uptake.	Silencing the expression of survivin (inhibitor of apoptosis) reduced the cell viability of MDA-MB-231 cell line to 63%.	[125]

**Table 3:** Levels of clinical trials and pharmacological effects of natural polymer-based drug delivery systems.

**Polymer**	**Drug**	**Tumor type**	**Clinical Status**	**Outcomes**	**References**
PEGylated Chitosan and breast cancer specific monoclonal antibodies	Doxorubicin, paclitaxel	Breast carcinoma (MCF-7)	Phase II	Elevated cytotoxicity against MCF-7 as compared to L929 cells.	[136]
Chitosan with prostate specific membrane antigen	Docetaxel, siRNA (BIND-014)	Prostate cancer	Phase II	Increased sensitization of PC3 cells and decline in tumor progression.	[137]
Cationic cyclodextrin	siRNA	Solid tumor melanoma (Ribonucleotide reductase subunit M2)	Phase I	Toxicity limited by dosage and CALAA-01 adverse effects.	[138]
Hyaluronic acid	4-methylumbelliferone (4-MU)	Hepatocellular carcinoma	Phase I	Downregulation of *has2*, *hyal2* and cancer stem cell proliferation.	[140]
Sphingomyelin/cholesterol liposomes	Vincristine	Acute lymphoblastic leukemia	Phase II	Administered in combination with myelosuppressive agents to treat patients with non-tolerated peripheral thrombocytopenia.	[142]
Chimeric anti-HER2 monoclonal antibody (Margetuximab)	Monotherapy (no adjuvant addition)	Metastatic HER2-positive breast cancer	Phase III	Significant binding capacity to CD16A (FcγRIIIA) and subvert binding capacity to inhibitory FcγRIIB. (CD32B). Improved progression-free survival (PFS).	[146]

## LIST OF ABBREVIATIONS

ADP: Adenosine Diphosphate
BET: Brunauer-Emmett-Teller
C4BP: Collagen Type IV
CED: Convection-enhanced Delivery
CEMIP-2: Cell Migration-inducing Hyaluronidase 2
CHO: Chinese Hamster Ovary
CXCR: Chemokine Receptor
DNA: Deoxyribonucleic Acid
DOPE: Dioleoyl Phosphatidylethanolamine
DOTAP: 1,2-Dioleoyl-3-trimethylammonium Propane
EphA2: Erythropoietin-producing Hepatocellular Carcinoma Receptors
EPR: Enhanced Permeability and Retention
ER: Estrogen Receptor
FDA: Food and Drug Administration
FITC: Fluorescein Isothiocyanate
GSH: Glutathione
HA: Hyaluronic Acid
HER2: Human Epidermal Growth Factor 2
HT-29: Human Colorectal Adenocarcinoma
HUVEC: Human Umbilical Vein Endothelial Cell
iRGD: Cyclic Arginine-glycine-aspartic Acid
MCF-7: Michigan Cancer Foundation-7
MDR: Multidrug Resistance
MMPs: Matrix Metalloproteinases
MRSA: Methicillin-resistant *Staphylococcus aureus*
PANDER: Pancreatic-derived Factor
PD-L1: Programmed Death Ligand 1
PDGFR: Platelet-derived Growth Factor Receptor
PEG: Polyethylene Glycol
PR: Progesterone Receptor
RGD: Arginylglycylaspartic Acid
RNA: Ribonucleic Acid
RNAi: Short Hairpin RNA
siRNA: Small Interfering RNA
SLN: Solid Lipid Nanoparticles
VEGF: Vascular Endothelial Growth Factor
